# Exosomes molecular diagnostics: Direct conversion of exosomes into the cDNA for gene amplification by two-step polymerase chain reaction

**DOI:** 10.14440/jbm.2018.249

**Published:** 2018-07-25

**Authors:** Shabirul Haque, Sarah R. Vaiselbuh

**Affiliations:** 1 The Feinstein Institute for Medical Research, Northwell Health, 350 Community Drive, Manhasset, NY 11030, USA; 2 Division of Hematology-Oncology, Department of Pediatrics, Staten Island University Hospital at Northwell Health, Manhasset, NY 11030, USA

**Keywords:** Exosome, cDNA, PCR, single cell PCR, Exo-cDNA, exosomal mRNA transcript

## Abstract

Exosomes are cell derived lipid nanoparticle with a size of 30–100 nm in diameter, found in almost all biological fluids. The composition of the exosomes is mainly lipid, proteins, RNA, DNA, and non-coding RNAs. Currently, most available methods and commercial kits for exosomal-RNA (Exo-RNA) isolation have limitations and shortcomings. Small starting volume of exosomes and the use of extraction/filtration columns results usually insufficient yield of exosomal RNA after isolation. The majority of RNA contained in purified exosomes range in size from 15–500 nucleotides. Some RNA isolation kits are well suited for small RNA transcripts isolation but larger mRNA transcripts are hard to detect. For all of the kits, the cost prize per sample analyzed is very high. Our current method provides a novel way for direct conversion of exosomes into cDNA synthesis (Exo-cDNA) and subsequent gene detection by polymerase chain reaction (PCR). This method has several advantages compared to established available kits. No extraction column is utilized in this procedure which means total recovery of exosomal RNA with maximal yield. In addition, this method is fast and uses a minimal amount of lab supplies, thereby reducing the overall working costs. Our findings suggest that direct conversion of exosomes into cDNA and subsequent gene amplification by two step PCR is a most efficient and reproducible technique. This novel method can be applied to and is useful to advance molecular research of exosomes by solving the problem of low molecular yields.

## INTRODUCTION

Exosomes are spherical (lipid bilayer) nanoparticles secreted by almost all cell types which contain an endomembrane system. Exosomes are composed of proteins, DNA, mRNA and non-coding RNA [1-2]. The size of exosomes range between 30–100 nm, and they originated from the cytoplasm of the cell by inward budding of multi-vesicular bodies [3]. Exosomes are abundantly found in bio-fluids such as saliva, serum, urine, breast milk, cerebrospinal fluid, and amniotic fluid [4-7]. In addition, exosomes have been isolated *in vitro* from conditioned medium (CM) of cultured cell lines [8]. In biomedical research, understanding the nature of exosomes is especially important to facilitate early diagnosis, early relapse, risk stratification, and targeting of the disease by real-time exosomal biomarker studies. However, progress in exosomal biomedical research is still facing some hurdles to overcome. Although there are several commercially available methods for exosomal RNA isolation, most of them have some limitations. A ready-made pre-designed manufactured exosome isolation column can usual process up to 4.0 ml of serum, but processing smaller volumes of less than 1.0 ml of serum yields poor quality of exosomal RNA [9]. Processing of higher sample volume columns is also more expensive. In addition, the multi-step processing of the exosome samples first through the isolation column, followed by several washing steps to retrieve the exosomal RNA, results in loss of exosomal RNA. Other exosomal RNA isolation kits are based on the acid-phenol: chloroform extraction method to provide a robust front-end RNA extraction followed by a final RNA purification over a glass-fiber filter by addition of ethanol to the columns, to immobilize the isolated RNA. The filter is then washed for RNA elution but again, the step wise filtration/washing procedure results in loss of RNA yields [10]. Investigators in exosome research are still juggling for the optimal exosomal RNA extraction method that results in greater yields and higher quality of RNA from the smaller sample volume. There is a dire need to improve the sensitivity of existing exosomal RNA isolation methods while maintaining minimal usage of the already spare exosomal starting volumes. Haque *et al*. had previously described a RNA isolation method from single cells for detection of p53 mRNA transcript. In this study, single cells were sorted by flow cytometry into polymerase chain reaction (PCR) tubes, and then lysed by adding lysing buffer in the same PCR tube, with subsequent direct conversion of isolated RNA into cDNA. The p53 mRNA expression was confirmed by using the obtained cDNA template in a two steps PCR [11]. Since p53 is present at low copy number in the cell, two rounds of PCR amplification were necessary for detection in this so-called two step PCR method. Based on the successful implementation of the two step PCR method for RNA isolation from a single cell (which implies a very low RNA starting volume), we applied this method for exosomal RNA isolation as well. Extrapolation of the single cell RNA isolation method to exosomal research might have been a far stretch if we only compare the diameter size of a single cell (8–12 µm) with the diameter size of an exosome (30–100 nm) [12]. In addition, the copy number of mRNA transcript carrying capacity is also different in cells and exosomes. However, since purified exosomes are pooled together after isolation from body fluid, serum or CM and the exosomal start sample for RNA isolation is quantitated by protein content measuring (NanoDrop), we thought it might be worth trying.

This study focuses on the method of direct conversion of exosomes into cDNA and gene amplification by PCR. It has several advantages such as: (1) no use of an extraction column resulting in higher yields of exosomal RNA recovery; (2) smaller starting volume of exosomes is sufficient for the procedure; (3) less time consuming than existing methods; and (4) cheaper than commercially available kits and methods. Making this reproducible method available for widespread use in the scientific exosome research community might hopefully at least alleviate one of the hurdles in this field.

## MATERIAL AND METHODS

### Cell lines and cell culture

The acute lymphatic leukemia (ALL) cell lines SUP-B15 (cat. # ATCC^®^ CRL-1929™), and JM1 cells (cat. # ATCC^®^ CRL-10423™) were purchased from ATCC. SUP-B15 cells were cultured in Iscove’s Modified Dulbecco’s Medium (IMDM), supplemented with 1.5 g/L sodium bicarbonate, 0.05 mM β-mercaptoethanol, 20% fetal bovine serum (not heat inactivated), and 1× Penicillin-Streptomycin. JM1 cells were expanded and cultured in IMDM supplemented with 0.05 mM β-mercaptoethanol, 10% FBS, and 1× Penicillin-Streptomycin. Normal B-cell line cells CL01 (Chiorazzi lab, The Feinstein Institute) were expanded and cultured in RPMI-1640 supplemented with 10% FBS, and 1× Penicillin-Streptomycin. All cell lines were incubated in a humidified incubator at 37°C with 5% CO_2_. Solid tumors cell lines such as HepG2 cells (Hepatocellular carcinoma cell line), HeLa cells (Cervical cancer), and SiHa cells (Squamous cell carcinoma) were placed in tissue culture to isolate exosomes from conditioned medium.

### Collection of human serum

Healthy donor (HD) and ALL patients’ serum samples were obtained after informed consent according to Institutional Review Board (IRB) approved protocol. Serum was isolated from whole blood by the standard method of centrifugation. Age and gender of the human samples cannot be released for Health Insurance Portability and Accountability Act (HIPAA) protection as per institutional IRB guidelines.

### Exosome isolation by ultracentrifugation

Purification of exosomes was carried out by ultracentrifugation method [8]. In brief; CM from cultured cells or human serum was subjected to centrifugation for 10 min at 300× *g*. Supernatant was collected and centrifuged for 10 min at 2000× *g*. Again, supernatant was collected and centrifuged for 30 min at 10000× *g*. Then supernatant was ultra-centrifuged for 120 min at 100000× *g*. Pellets containing isolated exosomes were re-suspended in phosphate buffered saline (PBS) and ultra-centrifuged for an additional 120 min at 100000× *g*. Exosomes containing pellets were harvested and reconstituted in 500 µl PBS. Each centrifugation step was carried out at 4°C. The protein content of the exosomes was determined by a BCA protein assay kit (Bio-Rad) and NanoDrop ND1000 spectrophotometer. Isolated exosomes from CM (Exo-CM) and serum (Exo-serum) were aliquoted and stored at **−**80°C for further usage.

### Exosome isolation by column based kit

Alternatively, exosomes of human serum and conditioned medium of cells lines were isolated by a column based kit (cat. # 76064, Qiagen) as per manufacturer protocol.

### Protocol for Exo-cDNA (Exosomes into cDNA)

Details of the composition of buffers A, B and C are listed in **Table 1.** The source of reagents is mentioned below.

### Source of enzymes and inhibitors

RNase inhibitor (4 unit/µl) (cat. # 1055213, Qiagen), rRNasin RNase inhibitor (40 U/µl) (cat. # N251B, Promega), RT-Superscript III (200 U/µl) (cat. # 18080-044, Invitrogen), random hexamer primers (cat. # SO142, Thermo Fisher Scientific).

### Protocol steps

1. Take purified exosomes (40–200 µg protein in 10–12 µl volume PBS) into PCR tubes (USA Scientific).
2. Add buffer A (3.6 µl/reaction) into the same tube, mix by gentle tapping and place on dry ice for 5 min.
3. Transfer the PCR tubes from dry ice to wet ice and add buffer B (3.5 µl/reaction), mix well by gentle tapping and incubate for 5 min at 65°C.
4. Centrifuge (1000–1500 rpm for 10–20 s) the PCR tubes to collect samples at the bottom then keep samples on ice.
5. Add buffer C (7.0 µl/reaction) into the same tube, mix by gentle tapping and centrifuge to collect sample at the bottom.
6. Place PCR tubes with samples into thermo-cycler (Gene Amp PCR system, Applied Biosystems) programmed as follows: Reverse transcriptase (RT) steps are 42°C for 5 min, 25°C for 10 min, 40°C for 60 min, 70°C for 10 min (denaturation step).
7. Centrifuge the PCR tubes to collect samples at the bottom of the tube and store the samples containing the cDNA templates at **−**20°C for further use.

### Gene amplification utilizing Exo-cDNA as template by two steps PCR

Gene amplification of β-actin, HLA-DR, CXCR4 and CD34 was carried out by PCR. For PCR amplification, a total reaction volume of 30 µl was obtained by mixing 2.0 µl of Exo-cDNA. Briefly, first round of PCR was carried out using AccuPrime™ Pfx DNA Polymerase (cat. # 12344024, Thermo Fisher Scientific) (1 unit/reaction in 30.0 µl final volume). Second round of PCR was carried out using Ampli Taq Gold (1 unit/reaction in 30.0 µl final volume) (Applied Biosystems, Roche, NJ). Both cycles of PCR ran under the following cycling conditions: denaturation at 95°C for 30 s, annealing at 56°C–60°C for 60 s, and extension step at 72°C for 60 s for 35 cycles. Cell-cDNA (control) and Exo-cDNA (2 µl) were used as template for the first round of PCR. For the second round of PCR, 1–2.0 µl of the PCR amplicon of the first round was utilized as template, AmpliTaq Gold (Applied Biosystems, Roche, NJ) was added to a final volume of 30 µl and the thermo-cycler was programmed in the same way as for the first round of PCR. Primer sequence, gene accession numbers, annealing temperature, and amplicon size are listed in **Table 2**.

### Agarose gel electrophoresis

PCR amplified products were analyzed by agarose gel electrophoresis as follows: 1.5% agarose gel (cat. # CA3510-8, Denville Scientific, Inc.). Gel was prepared in 1× Tris-borate-EDTA buffer (cat. # 28355, Thermo Fisher Scientific). SYBR Safe (cat. # S33102, Invitrogen) was added to the agarose gel before polymerization. Agarose gel was loaded with 5.0 µl of each PCR amplicon product mixed with 6× loading dye (cat. # R0611, Crystalgen) and ran for 20–25 min at 200 V. Each agarose gel was loaded with standard 100 base pair markers/ladder (cat. # 65-0321, Crystalgen). The gel was exposed and captured for imaging using a Bio-Rad gel documentation system (Bio-Rad, Hercules, California). Intensity/densitometry of agarose gel band was measured by Image J.

### Cellular RNA extraction and cDNA preparation (used as controls)

Total RNA was extracted from SUP-B15, JM1, and CL-01 cells by Trizol reagent method (Invitrogen). Quality and quantity of RNA was analyzed by NanoDrop ND1000 spectrophotometer. Isolated cellular RNA (2–5 µg) was used for cDNA synthesis by qPCR by adding Oligo-dT primers (Invitrogen) and M-MLV reverse transcriptase (cat. # 28025-013, Invitrogen) as per manufacturer's protocol.

### Protein expression by Western blot

Cell lysates of cultured cells (CL-01, SUP-B15, *etc*.) and exosomal lysate of exosomes were prepared in RIPA buffer (cat. # RO278-50 ml, Sigma-Aldrich) supplemented with protease and phosphatase inhibitor (cat. # PI 78441, Thermo Fisher Scientific). Protein was estimated by Pierce BCA protein assay kit (cat. # 23227, Thermo Fisher Scientific). Equal amounts of protein (15–20 µg) were loaded for electrophoresis on 4 to 15% SDS-PAGE (cat. # 456-1083, Bio-Rad). Electrophoresed proteins were transferred to an immune-blot polyvinylidene fluoride membrane (Bio-Rad). Membranes were blocked with PBS-Tween (0.1%) with 5% non-fat milk for 1 h and incubated with primary antibodies overnight at 4°C, followed by HRP-conjugated secondary antibody (dilution 1:2000; Santa Cruz Biotechnology), and then immunoblots were developed with enhanced chemiluminescent solution (Pierce, Rockford, IL). Primary antibodies calnexin (cat# sc-23954, Santa Cruz Biotech) and CD63 (cat. # 556019, BD Pharmingen) were used at 1:1000 dilutions. The blots were developed with a chemiluminescence detection kit (Pierce) and exposed to X-ray film (Eastman Kodak Co., Rochester, NY).

## RESULTS

### Characterization of exosomes by western blot and NanoSight (NTA) analysis

**Figure 1.**
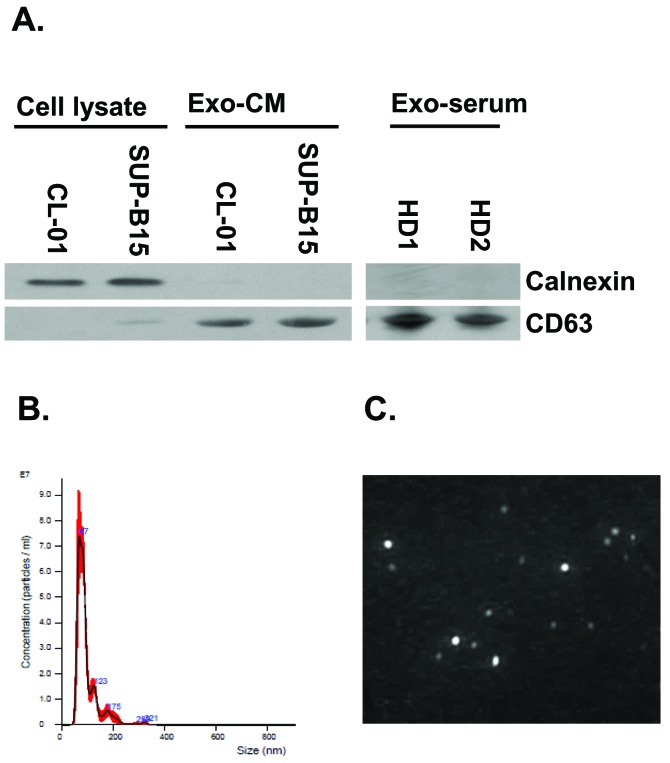
Characterization of purified exosomes. **A.** Western blot analysis for CD63 and calnexin of Exo-CM and Exo-serum. **B.** Nano Track Analysis (NTA) of isolated serum exosomes. Exosomal identity was confirmed for size and homogeneity. **C.** NTA exosomes imaging with representative picture of exosomes.

Exosomes were purified from both human serum and cell cultured supernatants/ CM by ultra-centrifugation. We checked expression of CD63 (exosome positive marker) and calnexin (exosome negative protein marker). Western blot shows that human Exo-CM and Exo-serum are negative for calnexin and positive for CD63 expression (**Fig. 1A**) which confirms the identity of the exosomes. We also examined and confirmed the size of the exosomes by nano-tracking analysis (NTA, Malvern). NTA confirms the correct size of the isolated exosomes (50–200 nm) (**Fig. 1B**), as well as provides an image of exosomes (**Fig. 1C**). As illustrated in the graph of **Fig. 1B**, it appears that 90% of exosomes are within the 50–100 nm diameter which is within the optimal range of size for exosomes and confirms their homogeneity.

### Amplification of exosomal β-actin by two step PCR

**Figure 2.**
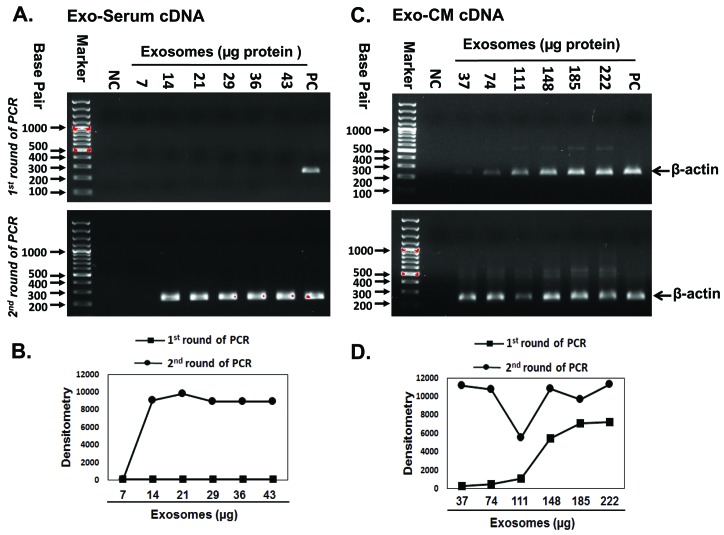
Amplification of exosomal β-actin by two-step PCR. Agarose gels were ran after first and second round of PCR amplification as shown in upper and lower panel respectively. **A.** Exo-serum cDNA (ALL patient serum) failed to amplify sufficient β-actin product after first round of PCR. **B.** PCR band intensity of (**Fig. 2A**) measured by image J densitometry. **C.** Exo-CM cDNA (JM1 cell line) did show a detectable amount of β-actin amplicon after first round of PCR. **D.** PCR band intensity of (**Fig. 2C**) measured by image J densitometry. Both Exo-serum cDNA and Exo-CM cDNA β-actin amplicons were detected after second round of PCR. A minimal exosomal amount of 14–37 µg was sufficient to obtain a signal only after two rounds of PCR. NC: negative control, no template added; PC: positive control, cell-cDNA used as template.

To apply the single cell RNA isolation and two step PCR method in exosomes, we first tested amplification of the endogenous housekeeping gene β-actin by two steps PCR (**Fig. 2**). Exo-cDNA synthesized by direct conversion from 14–43 µg Exo-serum failed to amplify β-actin in the first round of PCR (**Fig. 2A**, upper panel). It was not until after a second round of PCR that we were able to detect a band corresponding to the amplified β-actin Exo-cDNA (**Fig. 2A**, lower panel). Exo-cDNA synthesized from 37–222 µg Exo-CM of the JM1 cell line successfully amplified β-actin in first round of PCR (**Fig. 2C**, upper panel) with confirmation in a second round of PCR (**Fig. 2C**, lower panel). The PCR band intensity data by Image J densitometry identified the concentration dependent increase in PCR products (**Fig. 2B-2D**).

### Amplification of exosomal CXCR4 by two step PCR

**Figure 3.**
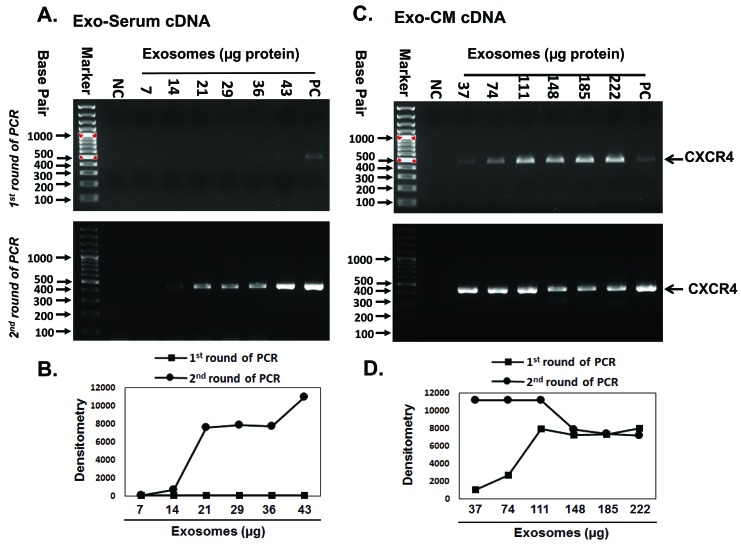
Amplification of exosomal CXCR4 by two step PCR. Agarose gels were ran after first and second round of PCR amplification as demonstrated in upper and lower panel respectively. **A.** Exo-serum cDNA (ALL patient serum) failed to amplify sufficient CXCR4 product after first round of PCR. **B.** PCR band intensity of (**Fig. 3A**) measured by image J densitometry. **C.** Exo-CM cDNA (JM1 cell line) did show a detectable amount of CXCR4 amplicon after first round of PCR. **D.** PCR band intensity of (**Fig. 3C**) measured by image J densitometry. Both Exo-serum cDNA and Exo-CM cDNA CXCR4 amplicons were detected after second round of PCR. A minimal exosomal amount of 14–37 µg was sufficient to obtain a signal only after two rounds of PCR. NC: negative control, no template added; PC: positive control, cell-cDNA used as template.

Next, we chose to use the CXCR4 gene for further verification of this method (**Fig. 3**). Exo-cDNA synthesized from 14–43 µg Exo-serum failed to amplify CXCR4 in a first round of PCR (**Fig. 3A**, upper panel). Detectable amplification of CXCR4 expression was found after a second round of PCR (**Fig. 3A**, lower panel). Exo-cDNA synthesized from 37–222 µg Exo-CM (JM1) successfully amplified CXCR4 in first round of PCR (**Fig. 3C**, upper panel) and second round of PCR also showed CXCR4 amplification (**Fig. 3C**, lower panel). It appears that CXCR4 is abundantly expressed in JM1 cell line-derived exosomes. The PCR band intensity data by Image J densitometry identified the concentration dependent increase in PCR products (**Fig. 3B-3D**).

### Two step PCR in Exo-cDNA and Cell-cDNA of parental cells

Exosomal RNA content is derived from the parental cell of origin. Therefore, it would make sense to test the two step PCR method on Exo-cDNA and compare it to Cell-cDNA analysis derived from the parental cell. Both templates should show amplification of the tested genes if exosomal content is indeed derived from the parent cell.

**Figure 4.**
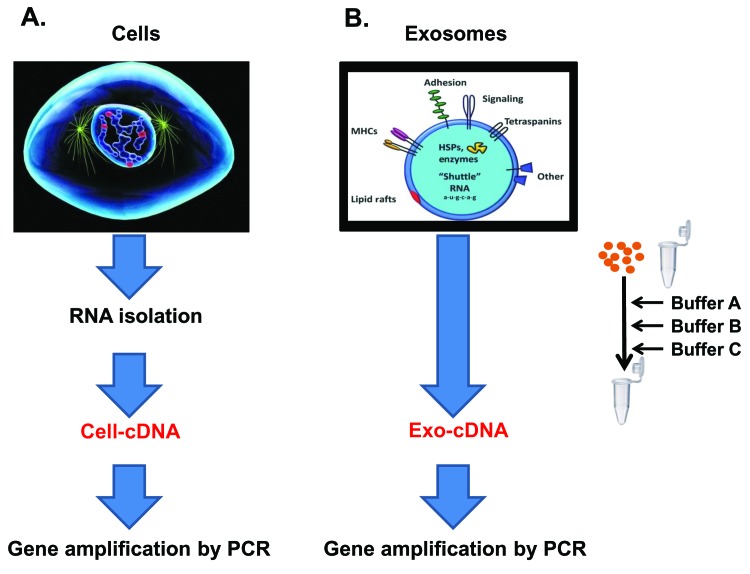
Schematic illustration of two step PCR in Cell-cDNA and Exo-cDNA of parental cells. **A.** Total RNA was isolated from parental cells by RNeasy Mini kit and Cell-cDNA was prepared by M-MLV reverse transcriptase kit (Qiagen). **B.** Purified exosomes were directly converted into Exo-cDNA by the direct conversion method (details are in material and method section).

Total RNA was isolated from cultured cells and Cell-cDNA was prepared by M-MLV reverse transcriptase kit (**Fig. 4A**). In contrast, purified exosomes of CM of the same cell line source in culture were harvested and processed according to the described direct conversion method. Briefly, after lysis by addition of a mild detergent, cDNA synthesis buffer and RT enzymes were added in the same PCR tube to achieve conversion of RNA into the cDNA (Exo-cDNA) (**Fig. 4B**).

### Amplification of HLA-DR and CD34 in Exo-cDNA compared to Cell-cDNA

**Figure 5.**
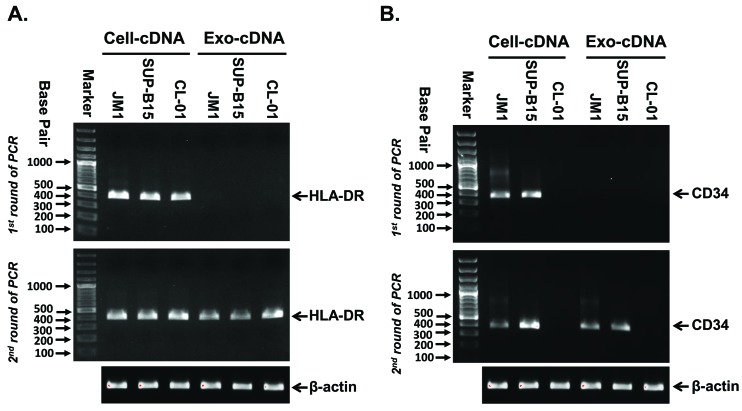
Amplification of HLA-DR and CD34 in Cell-cDNA compared to Exo-cDNA. Agarose gels were ran after first and second round of PCR amplification as shown in upper and lower panel respectively. **A** and **B.** Cell-cDNA for HLA-DR and CD34 amplified by a first round of PCR already in leukemia cell lines JM1 and SUP-B15, but CL-01 (B-cells) Cell-cDNA and Exo-cDNA remained negative for CD34 as expected. Exo-cDNA obtained from CM of leukemia cell lines only amplified HLA-DR and CD34 after a second round of PCR (β-actin housekeeping gene as quality control).

Three different cell lines (JM1, SUP-B15, and CL-01) were grown in tissue culture for harvesting of Cell-cDNA and Exo-cDNA of CM. HLA-DR and CD34 are both clinically used as markers for ALL cells; subsequently, Cell-cDNA and Exo-cDNA (2.0 µl) of ALL cell lines JM1 and SUP-B15 were compared as a template for amplification of HLA-DR and CD34 by two step PCR. CL-01 cells (B-cell line) do not express CD34 and was used as negative control (**Fig. 5**). In the first round of PCR for the HLA-DR gene, Cell-cDNA produced detectable amount of HLA-DR product but Exo-cDNA failed to amplify detectable amount of HLA-DR product on an agarose gel (**Fig. 5A**, upper panel). After a second round of PCR, both Cell-cDNA and Exo-cDNA produced detectable amount of HLA-DR product on an agarose gel (**Fig. 5A**, lower panel). The same pattern repeated itself for CD34 amplification: after the first round of PCR, Cell-cDNA of JM1 and SUP-B15 produced detectable amount of CD34 while Exo-cDNA did not amplify detectable levels (**Fig. 5B**, upper panel). However, after the second round of PCR, both Cell-cDNA and Exo-cDNA of JM1 and SUP-B15 produced detectable amount of CD34 copies while both cell-cDNA and Exo-cDNA derived from CL-01 cells remained negative for CD34 expression as expected (**Fig. 5B**, lower panel). Housekeeping gene β-actin was used as control for quality confirmation of Cell-cDNA and Exo-cDNA.

### Exo-UC and Exo-Col purified exosomes converted into Exo-cDNA for gene amplification

**Figure 6.**
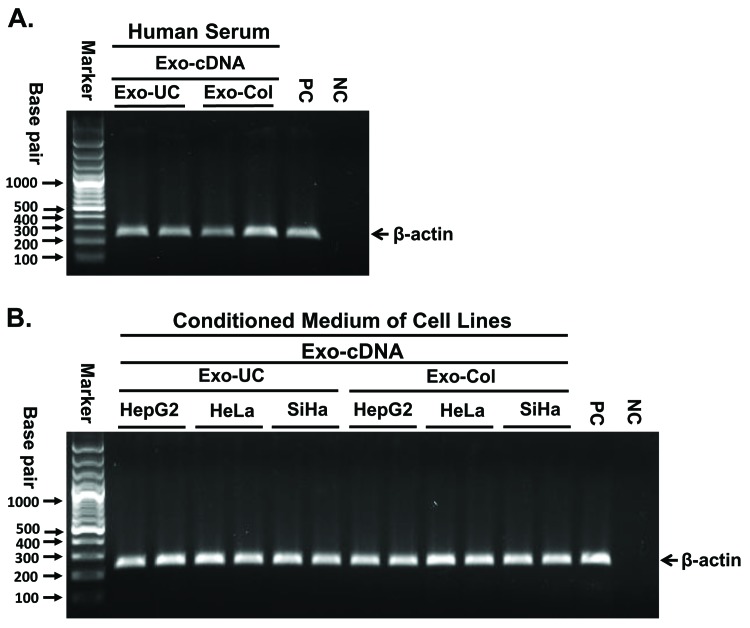
Exo-UC and Exo-Col purified exosomes converted into Exo-cDNA for gene amplification. **A.** Exosomes isolation from healthy donor serum samples by ultracentrifugation method (Exo-UC) and column-based commercial kit (Exo-Col) both converted into Exo-cDNA. PCR was carried out to amplify β-actin as shown on agarose electrophoresis. **B.** Exosomes were derived from the conditioned medium of three solid tumors cell lines (HepG2, HeLa, and SiHa) by both isolation methods Exo-UC and Exo-Col. Exo-cDNA was prepared from both exosomes sources and PCR was carried out to amplify β-actin as demonstrated on agarose gel image. NC: negative control, no template added; PC: positive control, cell-cDNA used as template.

To test whether our method works independently of how exosomes are isolated, exosomes were isolated by either ultracentrifugation (Exo-UC) or column-based commercial kit method (Exo-Col). No difference was identified between the two isolation methods which is useful when sensitivity is required with a low volume of samples. Both sources of purified exosomes were converted into the Exo-cDNA. Amplification of b-actin was carried out by PCR, following PCR product electrophoresed on agarose gel as demonstrated (**Fig. 6A**). In order to ensure the robustness of this method for multiple cell lines, we tested our protocol on a small panel of other cancers than leukemia. Exosomes were derived from the conditioned medium of three solid tumors cell lines (HepG2: hepatocellular carcinoma, HeLa: cervical carcinoma, and SiHa: squamous cell lung carcinoma) by both isolation methods Exo-UC and Exo-Col. Exo-cDNA was prepared from both exosomes source followed by PCR for β-actin amplification (**Fig. 6B**). Both Exo-UC and Exo-Col showed similar results confirming consistency and reproducibility of the method in other disease settings as well.

We tested our novel method in exosomes derived from both leukemia cell lines as well as solid tumors cell lines as mentioned in **Table 3.** We also tested within serum derived exosomes in 14 individuals (including healthy donors, leukemia diagnosed patients and relapsed patients) as listed in **Table 4**.

## DISCUSSION

**Figure 7.**
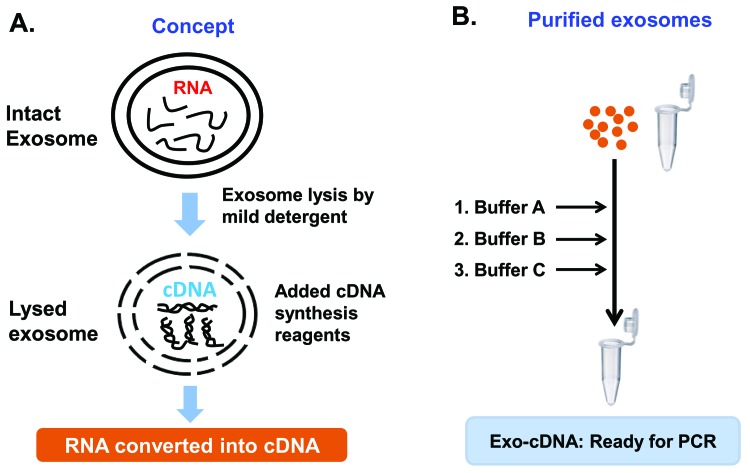
Schematic illustration of concept. **A.** Schmeatic diagram explains concept of the novel method, briefly intact exosomes memebrane were lysed by the addition of mild detergent which provides chance to access exosomal RNA for the conversion into cDNA (Exo-cDNA). **B.** Schmeatic diagram orients the steps of the protocol. Purified exosomes were taken into a tube and buffers A, B, C, were added into the same tube resulting Exo-cDNA as final outcome.

The structural nature of exosomes consists of a lipid bilayer surrounding the nano-sized particle. In this method, exosomes are treated with a mild detergent resulting in increased porosity of the exosomal membrane or exosomal membrane lysis. As a result, the exosomal content becomes available to interact directly with the reagents of the cDNA synthesis cocktail. All the RNA content gets converted into cDNA in the same tube at the same time (**Fig. 7**, panel A). Furthermore, we have successfully tested the Exo-cDNA obtained by this direct conversion method for downstream molecular analysis by nucleotide sequencing chromatogram (preliminary results—data not shown).

Exosomal RNA and DNA quantitation is emerging as a key in molecular diagnostics for biomarker development in pathological conditions, including cancer [13,14]. We have developed a novel method which allows for greater yields of RNA conversion to cDNA followed by two step PCR, resulting in enhanced sensitivity for detection of exosomal RNA. So far, existing methods involve first multi-step isolation of exosomal RNA (resulting in product loss) followed by conversion of exosomal RNA into the cDNA. Our current method is more compact and differs from other methods in that exosomes are lysed by mild detergent allowing for conversion of all RNA content directly into cDNA in the same tube in one single step. Utilizing this Exo-cDNA as a template, primer specific different genes such as β-actin, CXCR4, CD34, and HLA-DR were amplified by two step PCR. Exo-cDNA derived after direct conversion of RNA of exosomes at a minimal amount of 7–43 µg protein content did not yield a detectable band of amplicon after the first round of PCR. Only after a second round of PCR were we able to detect β-actin and CXCR bands on an agarose gel. When higher amounts of exosomes (37–222 µg) were converted into Exo-cDNA, a first round of PCR was efficient enough to amplify β-actin and CXCR expression on an agarose gel. A second round of PCR only showed more abundant amplification of both genes. The sensitivity of PCR amplification depends on the initial exosome amount taken for cDNA synthesis. Based on our findings, the lower amount of initial exosomes quantity (ranging from 7–40 µg) was only able to produce amplification after two rounds of PCR while higher amounts of exosomal starting quantity (ranging from 40–222 µg) showed abundant amplification already after the first round of PCR. Detection of different exosomal RNA content depends on the basal expression level of specific RNA in different cell types. Some RNA transcripts are abundantly expressed while some shows extremely low expression/copy number in nature within single cells [15]. Moreover, detection and quantification of any specific mRNA transcript from exosomes depends on the molecular signature of the parental cell from which exosomes are derived. As such, exosomes represent the fingerprint of the parent cells [12,16]. Stevanato *et al*. described that only 1/10th of parental cell miRNA gets transferred to their exosomal offspring, *e.g.*, if a single cell has 100 copies of miRNA, than exosomes derived from this parent cell will contain 10 copies of miRNA [17]. This explains how challenging the task is to detect and quantitate exosomal RNA as bio-markers in normal and pathological cells because of the lower copy numbers present in exosomes. Stoichiometry of the transfer of RNA from the parental cell to the exosomes is poorly investigated, but we have evidence that transfer of parental RNA to the exosomal RNA varies from 1/5th to 1/15th. This mRNA transfer from parent cell to exosomes depends on the type of mRNA, but the exact mechanism is not fully understood (Haque S and Vaiselbuh S, unpublished data). In this study, we have tested and compared by semi-quantitative two steps PCR, amplification of HLA-DR and CD34 expression in Cell-cDNA and Exo-cDNA. Interestingly, both cDNA templates utilized and produced detectable amount of PCR product on agarose gel at the second round of PCR. This shows that, despite of a low copy number of mRNA transcript present in exosomes, cDNA amplicons are detectable using this novel and sensitive method of direct conversion followed by two steps PCR.

In conclusion, we have developed a scalable and cost-effective method for the rapid isolation and quantitation of all RNA content present in exosomes, regardless of size, which is applicable to small amounts of exosomal starting material. This novel method may open the door to exploration of the nature and function of the complex mixture of RNA present in exosomes. This method represents a major breakthrough for the potential usage of exosomes in molecular diagnostics.

## Figures and Tables

**Table 1.** Composition of buffers A, B and C.

Buffer A	1 reaction
10× PBS	0.25 µl
H_2_O	2.50 µl
DTT (100 mM)	0.4 µl
rRNasin Rnase inhibitor (Promega)	0.2 µl
RNase inhibitor (4 unit/µl, Qiagen)	0.25 µl
Total	3.6 µl
Buffer B	1 reaction
Random primers (300 ng/ µl)	0.5 µl
NP-40 (10%)	0.5 µl
H_2_O	2.25 µl
PrimRNAse inhibitor (Qiagen)	0.25 µl
Total	3.5 µl
Buffer C	1 reaction
5× first strand buffer	3 µl
H_2_O	1.0 µl
DTT (100 mM)	1.0 µl
dNTPs (10 mM)	1.25 µl
rRNasin RNase inhibitor (40 unit/ml, Promega)	0.25 µl
RNase inhibitor (4 unit/µl, Qiagen)	0.25 µl
RT-SuperScript III (200 U/µl)	0.25 µl
Total	7 µl

**Table 2.** Sequence of human primers.

Gene	Accession number	Sequences	Annealing temperature	Amplicon size
CXCR4	AY242129.1	For*: 5'-AATGAGGCCACAACAAACATCACA-3’ Rev*: 5‘-ATCCAGACGCCAACATAGAC-3'	58°C	464 bp
CD34	M81104.1	For: 5‘-AATGAGGCCACAACAAACATCACA-3' Rev: 5‘-CTGTCCTTCTTAACCTCCGCACAGC-3'	56°C	380 bp
HLA-DR	K01171.1	For: 5‘-ATCATGACAAAGCGCTCCAACTAT-3' Rev: 5‘-GATGCCCACCAGACCCACAG-3'	60°C	404 bp
β-actin	NM_001101.4	For: 5‘-GTCCTCTCCCAAGTCCACACA-3' Rev: 5‘-CTGGTCTCAAGTCAGTGTACAGGTAA-3'	60°C	240 bp

*For, forward primer, Rev, reverse primer.

**Table 3.** List of 6 cell lines used as source for exosome isolation.

SN	Exosomes-derived from cell lines	Cell line types	Experiment (*n*)
1	JM1	ALL B cells	4
2	SUP-B15	ALL B cells	4
3	CL-01	Normal B cells	4
4	K-562	CML	1
5	HepG2	Hepatocellular carcinoma	1
6	HeLa	Cervical cancer	1
7	SiHa	Squamous cell carcinoma	1

**Table 4.** List of serum samples as source for exosomes isolation.

SN	Serum samples for exosomes		Experiment (*n*)
1	HD77 serum		3
2	HD78 serum		2
3	HD79 serum		2
4	HD80 serum		2
5	P-ALL25 serum (PB)	D1 = Dx	1
6	P-ALL25 serum (BM)	D1 = Dx	1
7	P-ALL24 serum (PB)	second remission	1
8	P-ALL24 serum (PB)	relapse D1	1
9	P-ALL14C serum (PB)	second remission	1
10	P-ALL14c serum (PB)	relapse D1	1
11	P-ALL01 serum D1(PB)	D1 = Dx	1
12	P-ALL01 serum D29 (PB)	first remission	1
13	P-ALL05 serum (PB)	D1 = Dx	1
14	P-ALL02 serum (PB)	D1 = Dx	1
